# Multi-beam two-photon polymerization for fast large area 3D periodic structure fabrication for bioapplications

**DOI:** 10.1038/s41598-020-64955-9

**Published:** 2020-05-26

**Authors:** Christian Maibohm, Oscar F. Silvestre, Jérôme Borme, Maina Sinou, Kevin Heggarty, Jana B. Nieder

**Affiliations:** 1 Ultrafast Bio- and Nanophotonics group, INL - International Iberian Nanotechnology Laboratory, Braga, Portugal; 2 2D Materials and Devices group, INL - International Iberian Nanotechnology Laboratory, Braga, Portugal; 3 Departement d’Optique, IMT-Atlantique, Technopole Brest-Iroise, CS 83818, Brest, France; 4 Present Address: Center for Cooperative Research in Biomaterials (CIC biomaGUNE), Basque Research and Technology Alliance (BRTA), Donostia-San Sebastián, Spain

**Keywords:** Optics and photonics, Applied optics, Laser material processing, Biomedical engineering

## Abstract

Two-photon polymerization (TPP) is capable of fabricating 3D structures with dimensions from sub-µm to a few hundred µm. As a direct laser writing (DLW) process, fabrication time of 3D TPP structures scale with the third order, limiting its use in large volume fabrication. Here, we report on a scalable fabrication method that cuts fabrication time to a fraction. A parallelized 9 multi-beamlets DLW process, created by a fixed diffraction optical element (DOE) and subsequent stitching are used to fabricate large periodic high aspect ratio 3D microstructured arrays with sub-micron features spanning several hundred of µm^2^. The wall structure in the array is designed with a minimum of traced lines and is created by a low numerical aperture (NA) microscope objective, leading to self-supporting lines omitting the need for line-hatching. The fabricated periodic arrays are applied in a cell – 3D microstructure interaction study using living HeLa cells. First indications of increased cell proliferation in the presence of 3D microstructures compared to planar surfaces are obtained. Furthermore, the cells adopt an elongated morphology when attached to the 3D microstructured surfaces. Both results constitute promising findings rendering the 3D microstructures a suited tool for cell interaction experiments, e.g. for cell migration, separation or even tissue engineering studies.

## Introduction

3D fabrication approaches including electro-spinning, nano-imprinting, additive 3D printing of ceramics, metals and plastics together with other forms of bottom-up techniques, have revolutionized tissue and organ engineering, cell migration research and other applications in biomedical research^1–6^. Additionally, advanced light-induced material processing techniques have been developed including mask-less and rapid micro-fabrication and -machining, e.g. for surface structuring, ablation and modifications^7–10^. Belonging to this class of methods is direct laser writing (DLW) based micro-fabrication, where single-photon DLW can fabricate 2D and 2.5D type structures, while the inherent sectioning capability of multi-photon based DLW allows the fabrication of 3D microstructures^11–14^. DLW has shown versatility in the fabrication of high-quality micro-optical elements^12^, waveguides^15^, and micro-machines^16^. Laser-based manufacturing is capable of processing bio-compatible materials^17–19^. As an optical technique DLW is limited by optical diffraction. Therefore, achievable feature sizes relate to the wavelength of the light source used. The microfabrication resolution furthermore is governed by the material properties, including the polymerization or ablation thresholds. The combination of these two aspects ultimately may allow for fabricating feature sizes well below the optical diffraction limit^20–22^. The DLW-based polymerization fabrication process is based on tracing the contours of the structure design in a photosensitive material, followed by a development step to remove the developed/undeveloped polymer to obtain the final microfabricated structure. As a serial process, the fabrication time of DLW power scales with the dimensionality of the structure, i.e. to the third power in 3D. This might not present a limiting factor in case of small – high quality structure fabrication, but can hinder upscaling, when considering fabrication of large volume structures. Even though as a benchmark study, recently centimeter size structure fabrication using multi-photon DLW has been demonstrated^23,24^. For the fabrication of small features high NA optics are chosen, which create the needed small writing voxels. Large volume structures are therefore associated with long fabrication times requiring multiple tracing of closely spaced lines in X and Y, called hatching, as well as close layer separation in Z, called slicing. To compensate for the increase in fabrication time for the DLW process at high resolution/small feature size, two strategies can be employed. First, the writing speed, translation of the voxel through the photosensitive material, could be increased. Second, the light field at the focus, could be modified in a way that several voxels are created, thereby effectively transforming the serial into a parallel fabrication process.

For increased voxel movement speed the translation speed of stage scanning systems is typically the limiting factor for this approach, while inside the field-of-view (FOV) of the focusing objective the voxel speed can significantly be increased by using galvo-scanning mirrors^12^. Though, for larger structures extending beyond a single FOV stitching of structural elements becomes necessary^25^.

The parallelization of the DLW process can be achieved using holographic methods, either based on using a fixed diffractive optical element (DOE) or adaptive optical elements such as a spatial light modulators (SLMs) or micro-mirror arrays, or via splitting the excitation beam via lenselets into multiple excitation beamlets^13,26–30^.

A SLM or micro-mirror array gives the benefit of changeable patterns, even allowing for dynamic pattern changes during the fabrication process. However pattern quality is limited by the SLM pixel or mirror number, shape and density.

Common for DLW-based microfabrication, and especially important for TPP-DLW, where each photon carries energy below the polymerization threshold, is that the energy in each beamlet or pixel, is above the polymerization threshold. The writing speed and especially the maximum number of active voxels is therefore limited by the available laser power. Recent developments in high pulse power kHz repetition rate lasers and micro-mirror arrays have made it possible to reach massive parallelization of the TPP process with up to a million pixels^29,30^.

Here, we use a fixed beamlet pattern, which is well-suited for the fabrication of repetitive periodic structures with constant pitch, as these can be optimized to achieve high beamlet uniformity and quality. We demonstrate a scalable fast fabrication strategy for large volume continuous 3D structures containing sub-micrometer features. Such feature sizes are found in biology, where extracellular matrices contain networks with few micrometer fibrous structures^31^, while individual cells typically reach length scales of tens of micrometers. It has been shown that microstructured surfaces have an impact on cell polarization, motility, morphology and proliferation^32^. Importantly, 3D polymer microstructures may act as scaffolds for tissue engineering^33^. Various emerging research approaches make use of 3D polymer microstructures to develop an increased understanding via *in vitro* studies in various areas of medical research relevance, concretely in the area of cancer^31,34,35^, neuronal^25,36–38^, primary/stem cell research^39–45^ and even reached *in vivo* applications^46^.

Typically, extended structures – covering several FOV – are needed to host a relevant number of cells interacting with the 3D microstructures under identical conditions. Therefore, the fabrication time of microstructures for cell motility/interaction studies can be considered a relevant factor and reducing it may be considered important for a widespread technology uptake in cellular biology research and bioengineering.

Our approach relies on the combination of a fixed DOE for parallelized multi-beamlet writing with a relatively low NA microscope objective, to allow unit structure fabrication without hatching and reduced slicing.

We aim to reduce fabrication duration by 9 times using a fixed DOE designed to create 9 identical beamlets in a 3 × 3 pattern with a 50 µm pitch, matching to the size of the designed unit structures. We characterize the polymer microstructures using confocal fluorescence microscopy and high-resolution SEM imaging. Stitching of the 3 × 3 patterns is used to create large continuous structure arrays suited for cell scaffolds or tissue engineering and we show initial cell – 3D microstructure interaction results.

## Results

**Figure 1:**
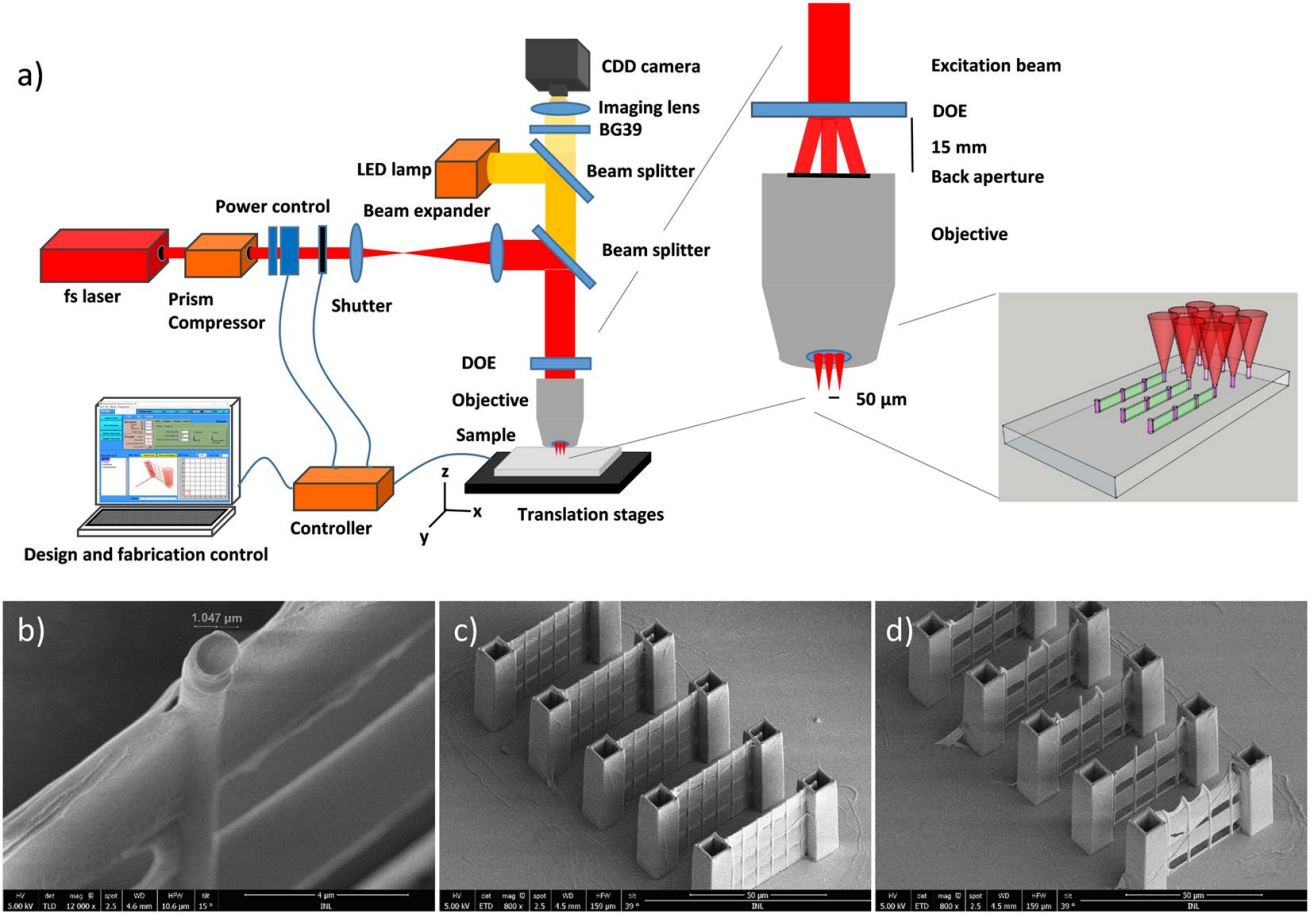
(**a**) Schematic representation of the TPP experimental setup based on a stage scanning system and a femtosecond laser; an enlarged cross section view of the beamlet-creating DOE and microscope objective as well as a 3D visualization of the parallelized writing using the 9 beamlets in the polymer. (**b**) SEM micrograph of a 3D structure detail, featuring a vertical line with a diameter of 1.047 µm (Z-direction), the dimension in the XY-plane is indicating that the lateral voxel size is well below 1 µm. (**c**,**d**) Study of voxel extend in the Z-direction. The horizontal line separation is changed from 7 µm (**c**) to 10 µm (**d**). For 7 µm the wall is nearly intact, while for a separation of 10 µm the wall becomes fully porous. We can estimate the voxel in Z-direction extension to be approx. 7 µm.

We fabricate 3D microstructures using a custom-designed TPP setup, based on a femtosecond laser guided into a stage scanning microfabrication work station. The custom design and accessible beam path allows the insertion of additional optical components, such as a DOE (see scheme in Fig. 1). The DOE creates 9 beamlets and the positioning and beam paths are indicated in an additional scheme, while 3D visualization indicates the writing process inside of a photosensitive polymer. First, we determine the voxel size based on the used microscope objective and fabrication parameters like stage scanning velocity and laser power, to be able to predict the resulting 3D polymer microstructures.

### Determination of the voxel size

The vertical and lateral dimension of the voxel are determined from fabricated vertical and horizontal lines for selected experimental parameters: a writing speed of 15 µm/s, a laser power of 11.5 mW and the SZ2080 bio-compatible photosensitive polymer.

We determine the lateral voxel size to be <1 µm for single traced lines in the XY- plane, while in vertical traced lines the voxel size reaches approximately 1 µm, as indicated by a marker in Fig. 1b. The slightly larger diameter of the vertical lines stems from additional polymerization caused by the comparatively larger vertical voxel extension leading to increased dose at same fabrication speed.

The vertical dimension of the voxel can be estimated from a 3D microfabricated structure that contains horizontal lines (see Fig. 1c,d). The chosen microstructure for voxel determination, contains a grid of vertical and horizontal lines connecting two larger towers. From the free hanging horizontal lines we can estimate the voxel in Z-direction. The line separation was varied from 5 (not shown) to 7 and 10 µm (see Fig c,d respectively) to identify the situation, when the lateral lines are no longer connected, resulting in small openings and horizontal cracks. This occurs at a separation of 7 µm, while at a separation of 10 µm between the horizontal lines, clear openings and a porous structure appears. Therefore, it is concluded that 7 µm is the vertical voxel size.

To test DOE created multi bealet based parallelization of the microfabrication process for large continuous arrays, we compare two different 3D designs. In both designs, the dimension of the unit structure is accurately matching the distance between the beamlets, created by the fixed DOE. Both 3D microstructure arrays consist of a single layer of unit structures with an XY extension of 450 × 450 µm^2^, created by stitching several 3 × 3 microstructures. The two designs of stitched structures can be divided into two broad categories of designs; structures with supported or unsupported stitching between individual structures. While different in height and number of walls, the two designs are such that they have the same wall surface area per unit structure.

**Figure 2:**
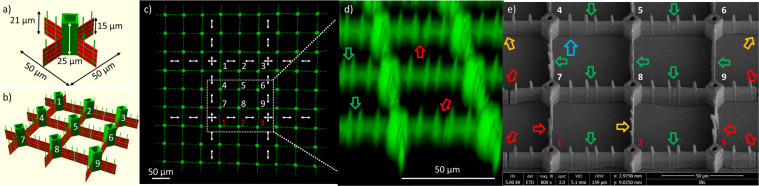
3D unsupported test structure for multi-beam writing. (**a**) 3D visualization of the unit structure, showing traced lines in green and voxel extension in the Z-direction in red. (**b**) 3D representation of the DOE replication of 9 identical unit structures. (**c**) Confocal fluorescence image of the full structure, where white arrows indicate the stitching between different fabrication replications. A white dotted square indicates the highlighted region in (**e**) where white numbers (1–9) indicates structures from one DOE replication, while red numbers (1–3) are unit structures from another DOE replication. (**d**) Tilted 3D confocal fluorescence image with arrows indicating error -free stitching in green and incomplete stitching in red. (**e**) Tilted-view SEM micrograph of the unsupported stitching. Green arrows indicate complete stitching inside the same DOE replication, while orange and red arrows indicate complete and incomplete stitching between structures from different DOE replications, respectively. One example of the sub-micrometer feature created by the difference between horizontal and vertical lines is indicated by the blue arrow.

The first design, categorized as structures with unsupported stitching, contains towers and walls, and stitching is meant to occur at the end facets of the walls. A perspective view of a 3D visualization of the expected microfabricated structures, which takes into account the determined lateral and vertical voxel dimensions, that govern the feature size of the resulting microfabricated structures, can be seen in Fig. 2a. Each of the walls are constructed by merely tracing 4 lines, 2 horizontal and 2 vertical lines (green). The horizontal lines form a wall due to the extended voxel in vertical direction, created by using a low NA microscope objective.

The unit structure shall be replicated in parallel 9 times by the DOE inside the FOV to create a multiplet structure. In Fig. 2b, we visualize the multiplet taking into account the voxel dimensions.

To achieve homogeneous features for all 9 beamlets for the parallelized TPP microfabrication process, the alignment between the laser excitation beam and the DOE was first optimized (Fig. S1).

The fabricated wall structure has an aspect ratio of more than 15, with a height of >15 µm and a thickness of <1 µm. The additional vertical lines seem to improve mechanical stabilization and provide anchoring of the wall to the sample surface, thereby strengthening the otherwise weakly bound wall structures. We observe that without addition of these lines, the wall segments collapse or move and bend during the development phase (data not shown). Secondly, the vertical lines create sub-micrometre features on the otherwise smooth wall surface, potentially interesting for cell interaction studies. While the walls and vertical pillars are fabricated by the same voxel at the same power and stage moving speed, the asymmetric voxel geometry with a 11 times increased extension in vertical direction causes a higher excitation dose deposited in the polymer positions of the vertically fabricated pillars.

The design of unsupported high aspect ratio single line interconnections demands very high precision in the stitching fabrication step. To evaluate the stitching quality throughout the full array confocal microscopy imaging, based on the autofluorescence signal of the SZ2080 polymer, and higher resolution SEM was used. In the confocal images incomplete stitching leads to discontinuity in the fluorescence from the structures as seen in Fig. 2c,d. The SEM image in Fig. 2d further emphasizes this, where several stitching mismatches, leading to breaks at the stitching position, are highlighted. We define two kinds of stitching results in the periodic structure. One where the resulting walls are intact (green and orange arrows) and a second, where the wall is broken (red arrows). Between structures created within a single FOV with the beamlets in parallel, only intact stitching is observed even if the wall segments seems to buckle, e.g. different orientations of the wall segments seem to occur between the stabilizing vertical pillars or towers. This is especially observable in Fig. 2e, when following the contact line between the polymer and substrate in the bottom of the SEM image.

This indicates that the matching between the structure design and the DOE pitch are perfectly matched and that the 50 µm travel of the stages does not introduce enough misalignment to compromise the single line stitching. Less accurate stitching is observed when multiplets are stitched. Then, both incomplete (red arrows) and complete (orange arrows) stitching results are observed. This indicates that the 150 µm long travel of the stages between different replications can introduce a small misalignment, and fusing extended <1 µm thin wall segments reveals to be difficult.

**Figure 3:**
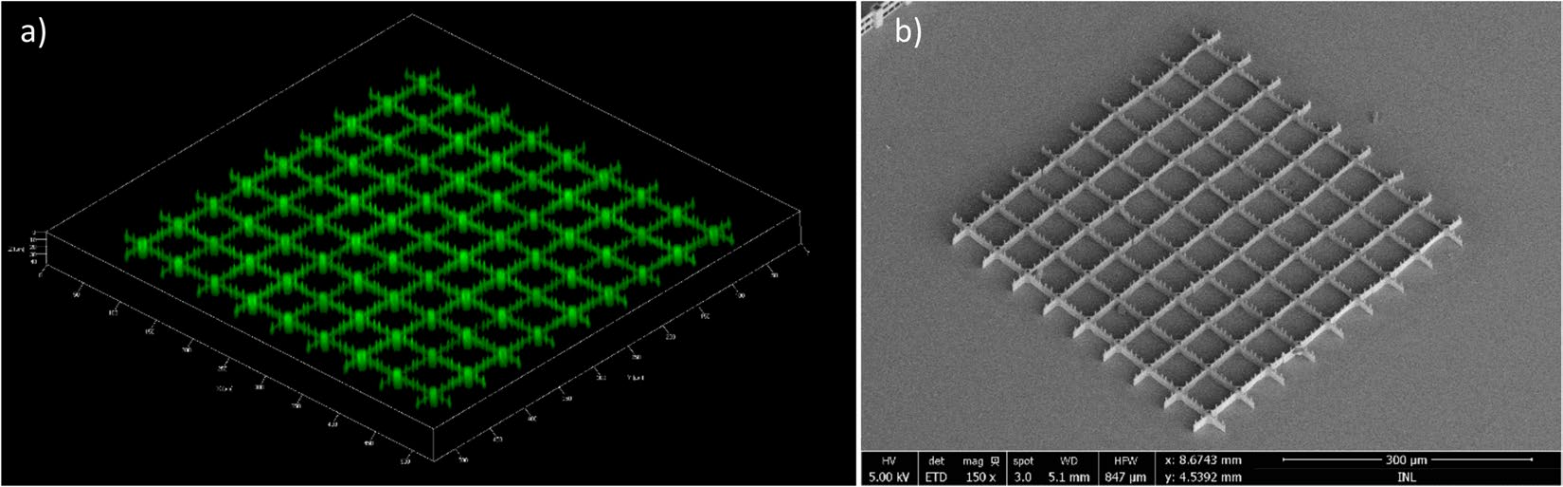
Array of unsupported stitched parallel fabricated unit structures. (**a**) Confocal fluorescence image of the full structure (20× objective). (**b**) SEM micrograph of the full structure covering an area of 450 × 450 µm, covered by a thin 10nm layer of gold. Fabrication time is 37 min for the full array where each individual unit structure takes 246 s.

In Fig. 3 we show a 3D confocal micro-fluorescence (a) and a SEM image (b) of the full structure covering an area of 450 × 450 µm^2^ containing 81 unit structures. The 3D sectioning confocal imaging allows to assess the 3D nature of the array and all structural features are well resolved.

To produce this extended array, only 9 unit structures are traced, and replicated 9 times. The parallel writing, using the 9 beamlets created by a fixed DOE, results in a 9 times shorter fabrication time.

Depending on the application of the microstructure stitching imperfection may hamper the intended functionality of the structure. One example would be the case of a photonic structure, where stitching discontinuities and structure imperfections would modify the periodicity or waveguiding properties and possibly render the structure non-functional. For most bio-applications the 3D microstructure requirements are expected to be less severe. Therefore, we conclude that using parallelized microfabrication reducing the fabrication time to a fraction via a holographic approach, paves the way for high throughput fabrication of 3D microstructures with bio-applications.

In the following, we aim to optimize the design of the unit structure for optimal stitching and chose extended features to be positioned at the stitching interface to overall reduce the number and extend of stitching errors.

### Optimizing 3D design for defect-free stitching

The second unit structure design for the stitching into a large area periodic structure is based on the conclusions made on the first design. The unsupported stitching between structures is changed to a supported structure. We exchange the single central tower with two towers, one at each end of a wall segment. Towers from two unit structures will fully overlap thereby creating a support between wall segments. The towers are 30 µm and the wall segments 27 µm tall, again fabricated by single line tracing. The distance between the horizontal lines is increased to 10 µm exceeding the vertical extension of the voxel. Therefore a porous structure emerges, as can be seen in the 3D visualization of the resulting unit structure in Fig. 3a. The size of the pores in the walls provides openings which lead to the possibility of potential cell passage and hence interesting opportunities for cell migration and cell separation studies^31^.

**Figure 4:**
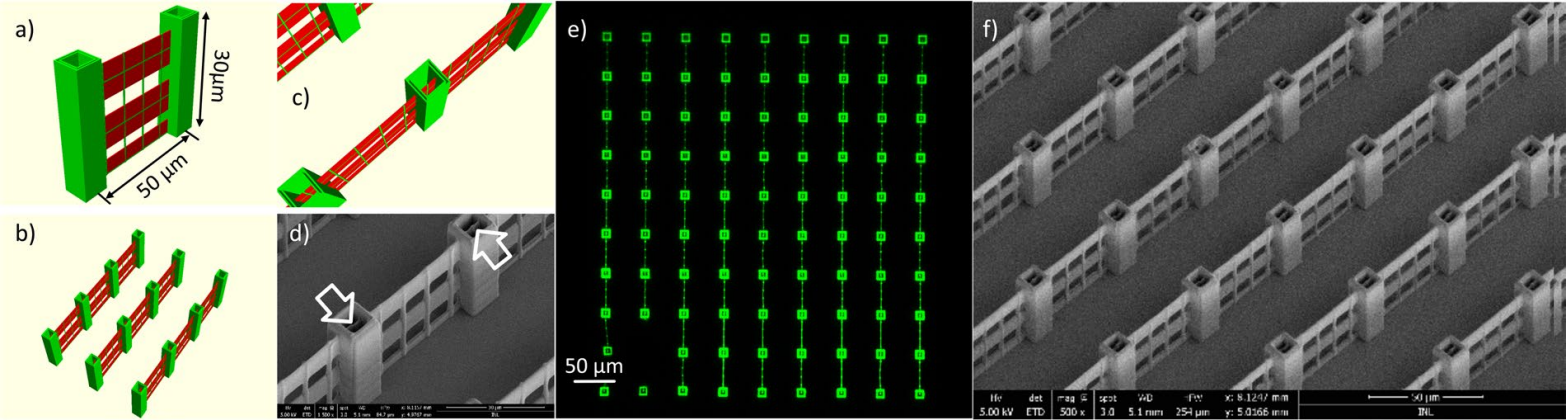
3D supported test structure for multi-beam writing. (**a**) 3D visualization of the unit structure, showing traced lines in green and voxel extension in the Z-direction in red. (**b**) 3D representation of the DOE replication of 9 identical unit structures. (**c**) 3D visualization highlighting the stitching between wall segments. (**d**) Tilted-view SEM image showing the towers with internal stitching positions, highlighted by white arrows. (**e**) Confocal fluorescence image of the extended array. (**f**) Tilted-view SEM micrograph of the DOE-written structure, across a larger area of the sample.

The unit structure is replicated in parallel 9 times by the fixed DOE shown in Fig. 4b, the wall segments are fabricated in one direction creating an open scaffold.

Stitching between wall elements from different structures occurs at the inside of the towers, as seen in the 3D visualization in Fig. 4c, and in the SEM micrograph of the fabricated structure shown in Fig. 4d,f. We do not observe stitching errors using confocal fluorescence microscopy (see Fig. 4e), where continuous fluorescence signal is observed along the walls connecting the towers for both type of stitching – either the stitching between the beamlets or the 3 × 3 multiples. Similarly, the high resolution SEM micrograph shown in Fig. 4f does not show fabrication errors due to stitching positioning mismatch.

The full array of 81 unit structures is fabricated in the same way as for the first design. The multiplication of the beam into the 9 beamlets allows the parallel fabrication of the unit structure, such that single tracing of the unit structure results in 9 seemingly identical replica.

**Figure 5:**
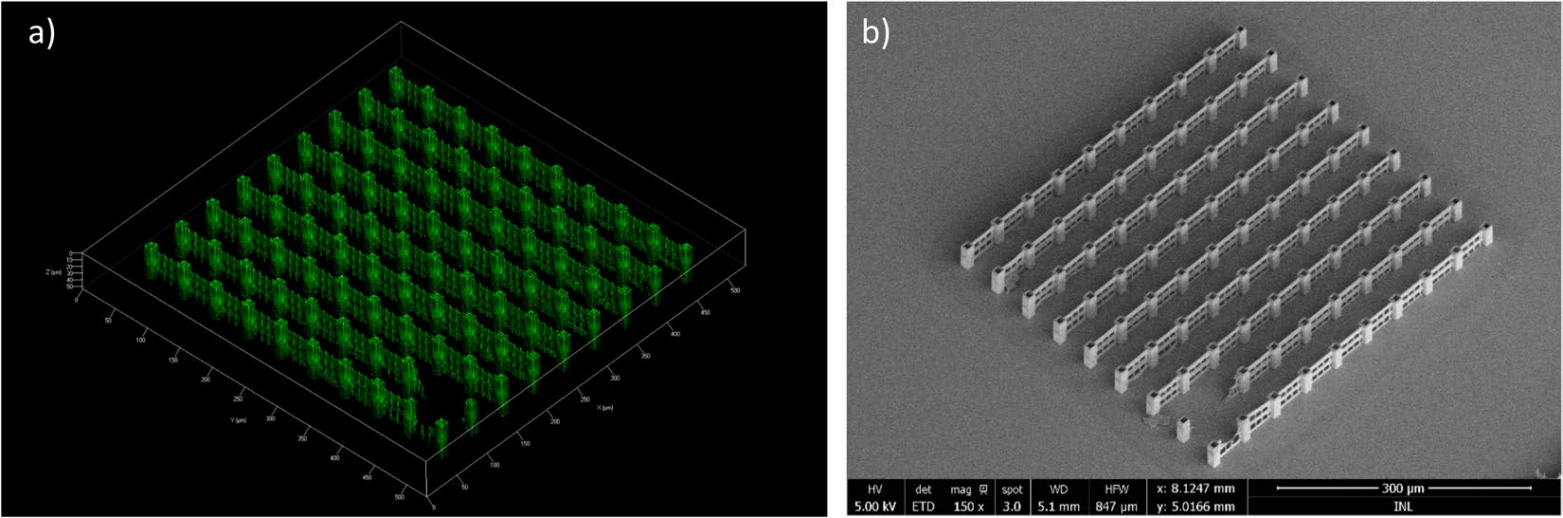
Array of supported stitched parallel fabricated unit structures. (**a**) Confocal fluorescence image of the full structure. (**b**) SEM micrograph of the full structure covering an area of 450 × 450 µm^2^ and with added thin 10 nm gold layer. Fabrication time is 56 min for the full array where each individual unit structure takes 373 s.

In the confocal microscopy image in Fig. 5a the fluorescence from the polymer is again used to map the 3D nature of the array and even details such as the porous wall can be characterized, which can also be observed in the tilted perspective SEM micrograph shown in Fig. 5b.

A small part of the array is collapsed, which happens occasionally in the development and subsequent drying process, especially for high aspect ratio structures. Each wall segment is straight, indicating that the supporting tower structures are indeed stabilizing the wall segments, leading to straight 40 µm spanning wall connections between the towers. The wall continues into the tower for another 5 µm, therefore after stitching of the unit structures an extended wall with a total length of 440 µm in the periodic array shown in Fig. 5 is obtained.

We show that parallelized TPP fabrication based on a holographic approach can be used for faster fabrication of extended 3D periodic structure arrays, while additionally we find that it is advisable to optimize the unit structure design for error-free stitching.

To demonstrate the potential of these extended 3D microstructure arrays for bioassays, and to provide an insight on the obtained feature sizes compared to live cells, we perform microstructure – cell interaction studies using confocal and optical transmission microscopy.

### Cell – 3D microstructure interaction studies

**Figure 6:**
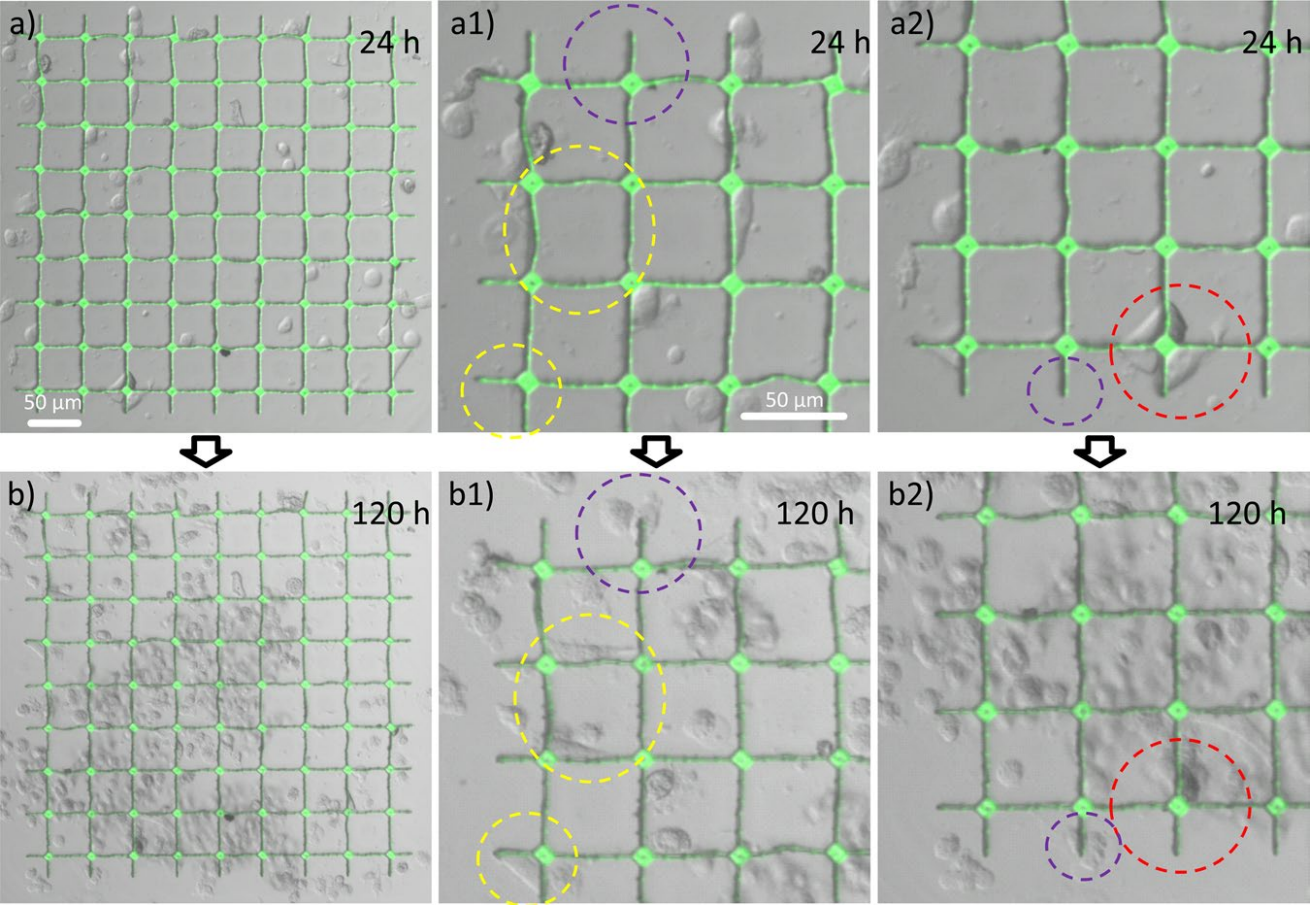
Combined confocal and optical transmission microscopy images of HeLa cells incubated on gold-coated 3D microstructures after 24 h (**a**), and zoom images in (**a1**,**a2**) and after 120 h in (**b**) with zoom into selected areas given in (**b1**,**b2**). We highlight areas in purple and yellow, where at 24 h of incubation no cells are present, while after additional 96 h of incubation, individual cells attach to walls or edges, respectively. The red circle indicates the opposite situation, where cells are attached to walls at 24 h of incubation, that relocate during the following 96 h leaving empty wall segments behind.

To evaluate the application of the periodic structures in the area of bioengineering and cell biology, we analyze the live cell – microstructure interaction over several days. We use HeLa cells, which are incubated on both structure arrays, and record confocal and transmission images after 24 and 120 h of incubation (see Figs. 6 and S2), respectively. For SEM characterization and to create a homogenous surface on the substrate, a 10 nm thin pure gold coating was added on the microstructured substrates. A thin continuous gold layer has been reported to be biocompatible and does not show inhibitory effects on cell growth after 72 h using fibroblast cells^47^.

Cultured cells in 2D receive different environmental cues compared to cells in natural tissues, and the potentially beneficial effect of 3D scaffolds to support mimic real tissue has been analyzed in various studies comparing 2D and 3D cell cultures^48,49^. To study the effect of 3D microstructures on HeLa cell behavior, we compare the cell population and morphology close to the 3D microstructures and on the 2D flat areas of the same sample (see Fig. S3).

The strong fluorescence from the SZ2080 photosensitive polymer is easily detected, while the positions and morphology of the cells is determined from the simultaneously recorded bright field images. The gold coating on the other hand ensures that the incubated cells experience no surface difference between the polymer structures and the glass substrate and the effect of the 3D microstructuring compared to flat 2D surfaces on cell behavior should therefore be directly observable from the experiments. For the first design, the interaction of the incubated HeLa cells and the 3D microstructure after 24 and 120 h of incubation is shown in Fig. 6. We highlight cells attaching to the scaffold after 24 h or 120 h of incubation, respectively and highlight specific dynamic changes, indicated by circles.

In the zoom areas shown in Fig. 6a1,a2 it is observed, that HeLa cells attach to both wall and corner segments, shown in yellow and purple, respectively. The cells change their morphology when interacting with the 3D microstructures and appear elongated between towers and even adopt triangular shape in the corners. We conclude that cells in the proximity of the 3D microstructures can alter their overall morphology compared to the typically more rounded or elliptically stretched cellular shapes present on the planar 2D surface (see Fig. S3a). Only a few cells in the area are not in direct contact with the scaffold, indicating a good affinity of the cells to the 3D microstructures. After 24 h of incubation, the number of cells inside the area of the scaffold is roughly the same as in a similar area on the planar 2D gold surface, indicating similar growth conditions.

The situation changes after 120 h of incubation (see Fig. 6b), where the number of cells inside the area of the scaffold is observably higher, compared to the number of cells in a similar area on the planar surface (see Fig. S3b). Some morphological changes occur for cells in contact with the scaffold (see Fig. 6b1,b2). The area highlighted by a red circle shows cells attached to the scaffold at the 24 h time point, which are not present at these locations after additional 96 h of incubation, indicating that these cells are alive and mobile. The higher number of cells in the 3D microstructured area hints towards two possible mechanisms; either increased cell affinity towards 3D microstructured surfaces, such that cells are attracted from the flat 2D areas into the 3D pattered areas, and/or an effect of the 3D microstructured environment on increased cell proliferation. Quantifying and separating these effects of the 3D microstructures on cell populations could be the aim of future dedicated cell interaction studies.

## Discussion

We analyzed the ability to speed up a nonlinear microfabrication process using a multi-beamlets generating DOE structure in the excitation beam path. For a first design without support structures at stitching positions, high similarity is observed between the individual unit structures inside each repetition (see Fig. 2e, green arrow), while at the stitching positions between different repetitions discrepancies are observed leading to gaps in the overall periodic pattern (see Fig. 2e, red arrows). The gaps arise from contraction of the polymer material during the development and subsequent drying process. It is related to large leapfrog movement of the stage system (>150 µm) between repetitions. In the second design, additional support structures at stitching positions were added to overcome this problem. Using this strategy, no gaps are observed and any remaining mismatch is less than 100 nm at the stitching positions. Cell interaction studies with the 3D microstructured arrays show cellular affinity, changed cell morphologies and potentially enhanced cell proliferation.

## Conclusions

In summary, we have demonstrated a scalable method for fast fabrication of large volume scaffold structures with the potential for cell and tissue engineering. We demonstrate how parallelization of the DLW process by a fixed DOE cuts the fabrication time to a fraction for large unit structures, while maintaining sub-micrometer features in each unit structure. To further lower fabrication time, each individual unit structure is designed to minimize the number of traced lines by omitting hatching. Large continuous 3D microstructures composed of stitched unit structures with supported and unsupported interconnections were fabricated with the parallelized TPP method. Unsupported stitching between unit structures leads to incomplete connections, while for supported stitching of unit structures, the extension from multiplets to extended periodic arrays is defect-free. While important for photonic structures, for bio-applications the advantages of fast fabrication may outweigh the need for high precision periodicity. The initial cell interaction studies indicate high cell affinity. Several locations around 3D TPP-fabricated features show the accumulation of cells that form contact with  the 3D microstructures leading to small cell aggregates. These suggest the 3D TPP microstructures are promising as a tissue engineering scaffolding system.

## Materials and Methods

### DOE fabrication

The binary phase Fourier DOE generates a 3 × 3 array of spots designed using an in-house Iterative Fourier Transform Algorithm (IFTA), with a three-stage optimization procedure to improve spot array uniformity^50^. The 10 × 10 mm DOE was fabricated in a 630 nm layer of S1800 series photoresist (MicroChem) on a glass substrate using a custom parallel direct-write photoplotter. The glass substrate was then laser scribed to dice it to dimensions compatible with the multi-photon system microscope.

### Two-photon polymerization (TPP) - based micro-fabrication

#### Structure design in 3D software

All presented structures are designed directly in the μFAB software from Newport. Since the design and fabrication software is the same, no additional software is used for slicing and hatching.

#### TPP setup

A stage scanning microfabrication writing station (µFAB, Newport), with a fixed focused laser beam is used. Translation stages in XY have a travel range of 100 mm with 1 nm resolution and minimum step size of 10 nm, while in the Z-direction the travel range is 4.8 mm with 20 nm resolution and minimum step size of 60 nm. The movement of the translation stages to trace the lines of the structures is controlled by a Newport XPS controller. A femtosecond laser (Tsunami, Spectra Physics, repetition rate 80 MHz) tuned to 795 nm with a pulse length of approximately 100 fs after the external prism compressor was used as light source for nonlinear polymerization. For power control a combined half-wave plate and Glan-laser polarizer is used. A fast shutter is used to control the beam on/off during the fabrication process. A beam expander is used to fully illuminate the DOE and also to overfill the back aperture of the microscope objective. All the presented structures are fabricated with a 40× Nikon air microscope objective (N40×-PF) with a NA of 0.75. The field-of-view (FOV) for the DOE is chosen to be a 150 µm square to limit optical distortion. The DOE mask is inserted in the excitation beam path at a distance of 15 mm from the back aperture of the microscope objective. Sample focusing and process inspection during fabrication was monitored via a CCD camera protected by a BG39 filter.

#### Experimental procedure for 3D microfabrication

Cleaned 1 mm thick glass microscope slides are heated to 100 °C for about 30 min on a hotplate to remove eventual water and solvent residues. 20 µL of the SZ2080, low-shrinkage hybrid organic-inorganic zirconium containing sol-gel polymer, is drop-casted on the glass slide and subsequently baked for another 30 min at 100 °C^51^. After baking, the sample is mounted in the µFAB writing station and the 3D microstructure designs are traced in the polymer layer. For both of the presented scaffolds fabrication speed is set to 15 µm/s. The laser power was set to 148 mW before the DOE element. With a ~70% efficiency of the DOE element, a power of 11.5 mW is estimated for each of the 9 individual beamlets. Development of the fabricated 3D polymer microstructures involves washing with a 1:2 solution of 4-Methyl-2-pentanone and 2-Propanol for 45 min and subsequent air drying.

#### Confocal microscopy imaging

Confocal imaging was performed with a Zeiss LSM 780 confocal microscope with the 488 nm excitation line of an Ar^+^ laser. In parallel, transmission bright field images were recorded. Experiments were performed with a 20× dry objective for the developed polymer structures, mounted pointing downwards to allow 3D sectioning of the structures within the working distance of the objective.

For *in vitro* cell bioimaging studies a 10× dry objective was used and imaging was performed through the 1 mm microscope glass slide.

#### SEM imaging

A thin 10 nm layer of gold was sputtered on the samples with a Kenosistec - UHV multitarget confocal sputter and SEM micrographs were collected with a FEI NovaNanoSEM 650.

#### Cell culture

The Penicillin-Streptomycin 100 × solution, 0.25% Trypsin-EDTA, and Phosphate-Buffered Saline (PBS) were supplied from Corning Cellgro. HyClone Fetal Clone III serum was obtained from GE Healthcare and Modified Eagle’s Medium (MEM) was purchased from Biowest. HeLa cell line was purchased from ECACC (Cat. No. 93021013), grown under standard culture conditions and seeded inside of two silicone insert wells on the TPP functionalized microscope slide in MEM culture media supplemented with 10% serum, 1% of penicillin-streptomycin, incubated under 5% of CO_2_ at 37 °C.

## Supplementary information

Supplementary information.
