# Supplementary material for: Multi-beam two-photon polymerization for fast large area 3D periodic structure fabrication for bioapplications
Source: Sci Rep. 2020 May 26;10:8740. doi: 10.1038/s41598-020-64955-9 (PMC7250934; doi:10.1038/s41598-020-64955-9)
Supplement: Supplementary file 1 — Supplementary information. [file 41598_2020_64955_MOESM1_ESM.docx]

**Supplementary material**

**Optimization of homogeneity between beamlets**

Prior to the presented experiments the uniformity of structures fabricated with the DOE was tested as a function of the lateral position of the excitation beam. The optical feedback – reflections of the laser on the DOE as observed with the CCD camera of the µFAB setup (not shown) allows associating a pattern to each DOE position, this was optimized for homogeneity for the 9 sub beam intensities. After SEM analysis a similar homogenous optical pattern was selected.

Optimization of the beamlet homogeneity as a function of the relative position between the laser excitation and the DOE (see Figure S1).


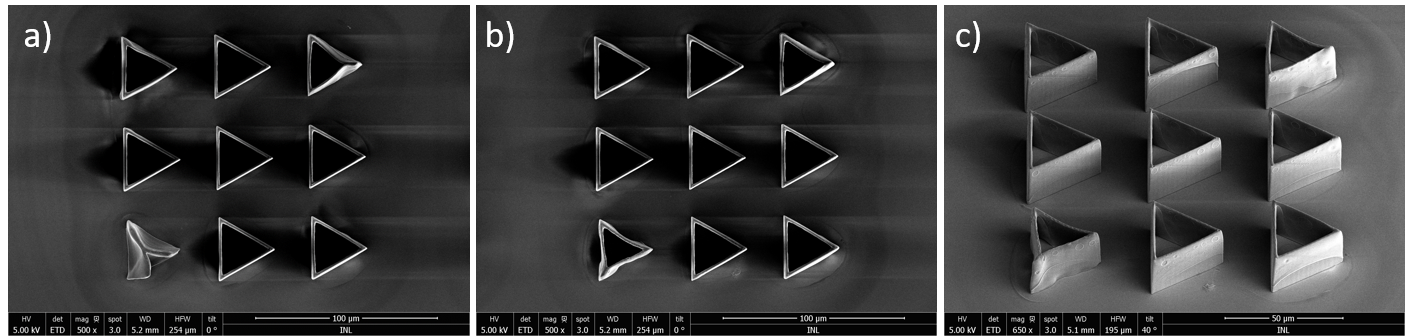


*Figure S1: The SEM images taken for the optimization process, optimizing the excitation beam position relative to the DOE, with an improvement from left to right.*

**Cell – 3D micro-structure interaction studies on 2^nd^ design**

For the second design, seen in Figure S2, the interaction of the incubated HeLa cells and the 3D micro-structures after 24 and 120 h of incubation is shown. It is observed that after 24 h HeLa cells attach to both wall segment and towers of the structures, highlighted in the zoomed sections in Figure S2 a1 and a2. The number of cells inside the area of scaffold is lower than on a similar area on the planar 2D reference area (see Figure S3 a). Yellow circles indicate areas, where cells attach to the 3D micro-structure after 120 h and show strongly elongated shapes.


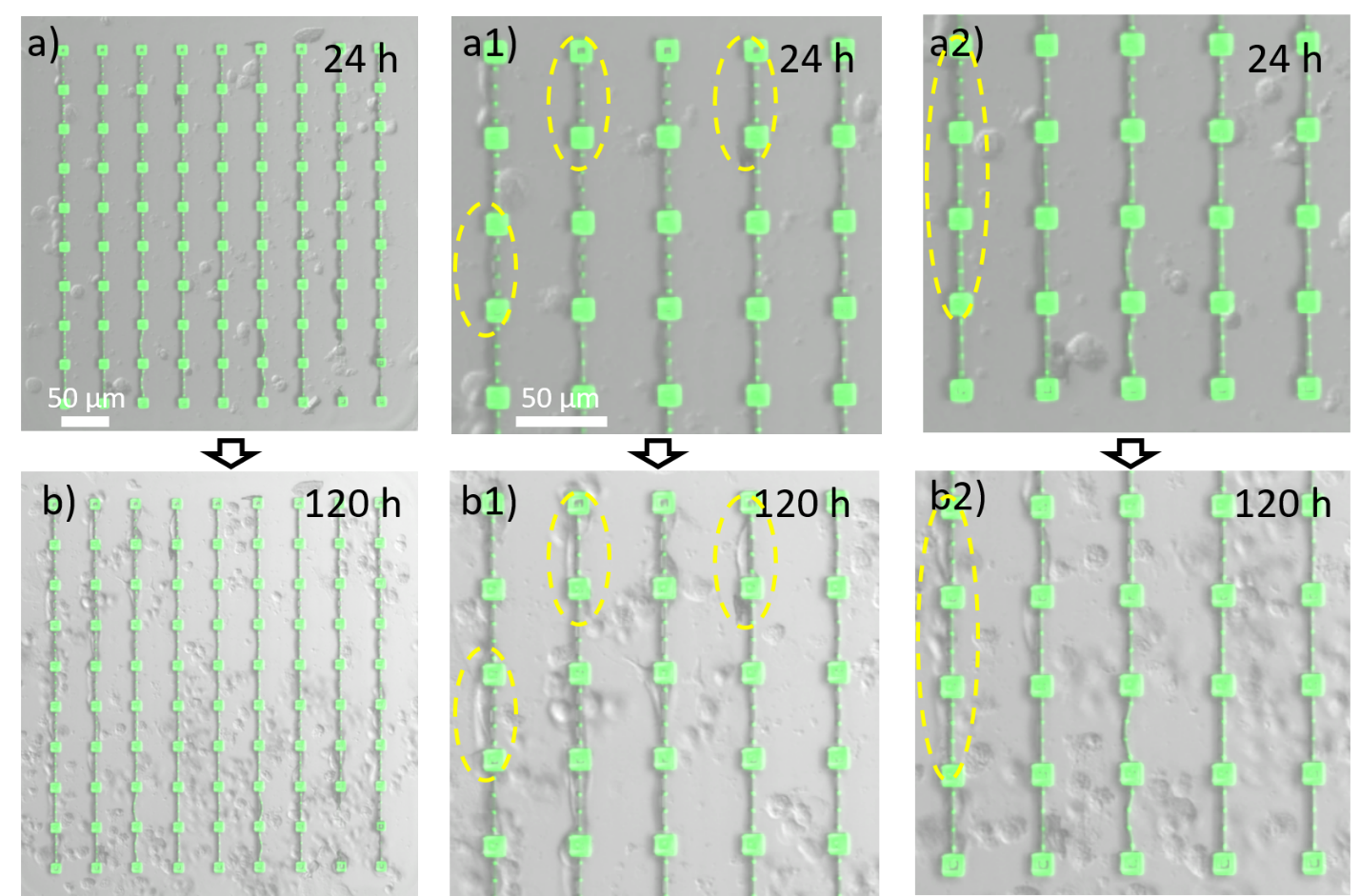


Figure S2: Combined confocal and transmission microscopy images of HeLa cells incubated on gold-coated 3D micro-structures after a) after 24 h with zoom images in a1) and a2 and b) after 120 h of incubation with zooms in b1) and b2) respectively. We highlight areas with yellow circles, that are empty at the 24 h time point, and which after additional 96 h of incubation, show presence of cells. These are found attached to the walls, and are of elongated shape.

After 120 h of incubation, the number of cells inside the area of the scaffold is observably higher compared to a similar area on the 2D planar reference area (Figure S3 b).


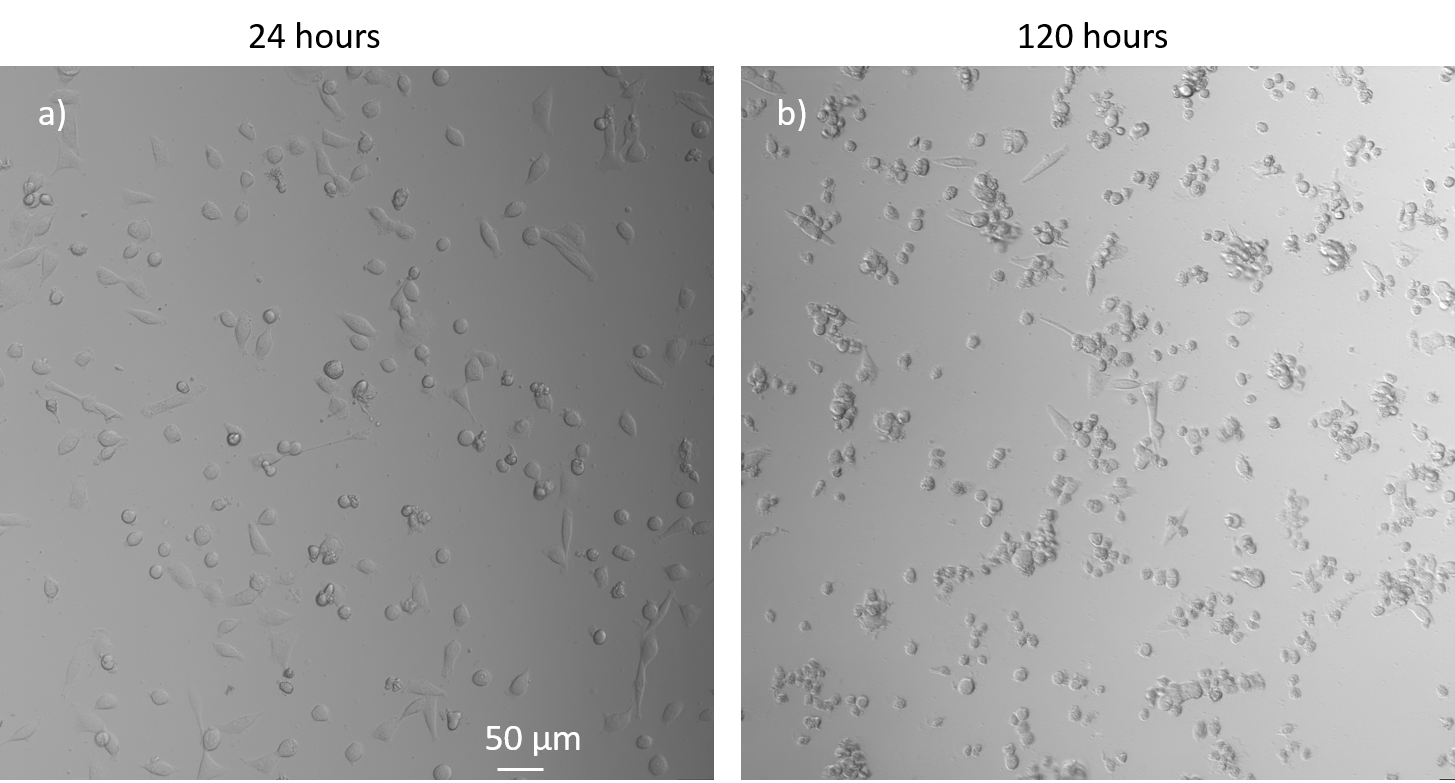


Figure S3: Bright field image of HeLa cells on the 2D planar areas of the sample after a) 24 h and b) 120 h of incubation used as reference for both type of 3D micro-structures.
